# The Application of Regulatory Cascades in *Streptomyces*: Yield Enhancement and Metabolite Mining

**DOI:** 10.3389/fmicb.2020.00406

**Published:** 2020-03-24

**Authors:** Haiyang Xia, Xiaofang Li, Zhangqun Li, Xinqiao Zhan, Xuming Mao, Yongquan Li

**Affiliations:** ^1^Institute of Biopharmaceuticals, Taizhou University, Taizhou, China; ^2^Institute of Pharmaceutical Biotechnology, School of Medicine, Zhejiang University, Hangzhou, China

**Keywords:** antibiotic production, regulatory cascades, rewiring regulatory network, unlocking cryptic metabolites, *Streptomyces*

## Abstract

*Streptomyces* is taken as an important resource for producing the most abundant antibiotics and other bio-active natural products, which have been widely used in pharmaceutical and agricultural areas. Usually they are biosynthesized through secondary metabolic pathways encoded by cluster situated genes. And these gene clusters are stringently regulated by interweaved transcriptional regulatory cascades. In the past decades, great advances have been made to elucidate the regulatory mechanisms involved in antibiotic production in *Streptomyces*. In this review, we summarized the recent advances on the regulatory cascades of antibiotic production in *Streptomyces* from the following four levels: the signals triggering the biosynthesis, the global regulators, the pathway-specific regulators and the feedback regulation. The production of antibiotic can be largely enhanced by rewiring the regulatory networks, such as overexpression of positive regulators, inactivation of repressors, fine-tuning of the feedback and ribosomal engineering in *Streptomyces*. The enormous amount of genomic sequencing data implies that the *Streptomyces* has potential to produce much more antibiotics for the great diversities and wide distributions of biosynthetic gene clusters in *Streptomyces* genomes. Most of these gene clusters are defined cryptic for unknown or undetectable natural products. In the synthetic biology era, activation of the cryptic gene clusters has been successfully achieved by manipulation of the regulatory genes. Chemical elicitors, rewiring regulatory gene and ribosomal engineering have been employed to crack the potential of cryptic gene clusters. These have been proposed as the most promising strategy to discover new antibiotics. For the complex of regulatory network in *Streptomyces*, we proposed that the discovery of new antibiotics and the optimization of industrial strains would be greatly promoted by further understanding the regulatory mechanism of antibiotic production.

## Introduction

*Streptomyces*, Gram-positive mycelial bacteria with high GC content, shows a complex morphological differentiation and belongs to actinobacteria (Hopwood, 2019). It is reported that 61% of so far discovered microorganism-derived bioactive substances are produced by actinobacteria (mainly *Streptomyce*). Most of the clinically essential drugs containing antibiotic and other bio-active agents are derived from *Streptomyces* (Waksman et al., 2010). In the past decades, scientists have made great advances to elucidate the regulatory mechanisms related to antibiotic production in *Streptomyces*. Generally, the antibiotic production is stringently and elaborately regulated by pyramidal transcriptional regulatory cascades, including signaling pathways, global regulators, pathway-specific regulator (PSR), and feedback regulation. This interweaved networks can determine the production levels of antibiotic under specific culture condition (Chater, 2016).

With the accumulation of genome data, the number of predicted secondary biosynthetic gene clusters (SBGs) on genome is much more than that of the products has been identified. Generally, most of these gene clusters have been defined as cryptic ones because of unknown or undetectable secondary metabolites. Scientists proposed that the *Streptomyces* has been greatly underestimated for the capability to produce diversity of natural products (Baltz, 2017, 2019). Up to now, *Streptomyces* strains are still taken as the most promising candidates for novel antibiotic discovery. In the synthetic biology era, manipulation of regulatory genes has been employed to activate of some cryptic gene clusters.

The production of antibiotic in *Streptomyces* can be largely enhanced by rewiring the regulatory network. There are several reviews on the advances about the elucidation of regulatory networks and the activation of cryptic gene clusters in *Streptomyces* (Ochi, 2017; Onaka, 2017; Wei J. et al., 2018; Palazzotto et al., 2019). Antibiotics or specialized metabolites usually means same for secondary metabolites produced by *Streptomyces*. Here, the term antibiotic is used to stand for the natural products. In this review, we will focus on the production enhancement and new antibiotic discovery by manipulation of the regulatory networks in *Streptomyces*. We proposed that the systematic rewiring of regulatory networks in *Streptomyces* would play a critical role in drug discovery and production enhancement in the near future.

## The Regulatory Cascades of Antibiotic Production in *Streptomyces*

It is commonly recognized that antibiotic production is stringently regulated by pyramidal transcriptional regulatory cascades in *Streptomyces*. Recent years, there were several reviews on the regulatory networks of *Streptomyces* (Romero-Rodriguez et al., 2018; van der Heul et al., 2018; Wei J. et al., 2018; Palazzotto et al., 2019). So here we won’t go into detail of the regulatory networks involved in antibiotic production. The regulatory cascades of antibiotic production will be briefly presented from the following four levels.

The first one is the onset of antibiotic production, which is triggered by the coupled receptors of *Streptomyces* “hormones” or other signals. Among them, A-factor, the chemical structural characteristic γ-butyrolactone (GBL), is the first *Streptomyces* hormone reported as a signal triggering streptomycin production in *Streptomyces griseus* in 1967. Avenolide was identified as a new type of butenolide hormone regulating avermectin production in *Streptomyces avermitilis* (Kitani et al., 2011). The production of the methylenomycin is induced by methylenomycin furan (MMF), a furan-type autoregulator in *Streptomyces coelicolor* (Xu G. et al., 2010). It was reported that 84.1% actinomycetes probably use either GBL (64.1%) or butenolide (24%) to control the antibiotic production (Thao et al., 2017). A-factor, coupled with its receptor ArpA, controls transcription of the master positive regulator AdpA for morphological differentiation and streptomycin production in *S. griseus* (Higo et al., 2012).

The second level is the global regulators which bring pleiotropic effects on the lower level of regulators. The global regulators affect more than one metabolic pathways and may not directly affect any specific biosynthetic gene clusters (BGCs). They also respond to a variety of chemical or physiological signals, e.g., nutrient limitation, the concentration of chitin or *N*-acetylglucosamine (GlcNAc) in the medium, cell wall damage, heat shock or pH shift. Two-component systems (TCSs, consisted with a membrane-bound histidine kinase, which senses specific environmental stimuli, and a cognate regulator) play crucial roles in sensing extracellular signals. Typical TCSs mediate responses to the cellular signal, mainly through regulating the transcription of downstream genes. Moreover, TCSs, as the most abundant pleiotropic regulators, are involved in the dynamic control of the biosynthesis of secondary metabolites in *Streptomyces*. E.g., PhoP/PhoR can regulate antibiotic production and morphological differentiation (Yepes et al., 2011; Rodriguez et al., 2013). Both antibiotic production and GlcNAc uptake *via* the phosphotransferase system are directly regulated by DasR, a GntR-family allosteric regulator, with the GlcNAc as a ligand (Fillenberg et al., 2016). The major checkpoint for the onset of secondary metabolism is the accumulation of GlcNAc through the autolytic degradation of the vegetative mycelium. The antibiotic production is triggered by DasR regulon (Rigali et al., 2008). AdpA, which is ubiquitously distributed in streptomycetes as a member of the AraC/XylS family regulators, affects transcription of hundreds of genes involved in morphological differentiation and antibiotic biosynthesis (Higo et al., 2012; Guyet et al., 2014). ArpA, the receptor of A-factor, regulated the transcription of *adpA* in *S. griseus*. The transcription level of *adpA* is dynamic controlled by the interaction between ArpA and A-factor. As A-factor reached a threshold concentration, it binds to ArpA and releases the repression of *adpA* transcription (Wei J. et al., 2018). WblA and AtrA also have been extensively investigated as the global regulators involved in the biosynthesis of antibiotic. As a global repressor, WblA affects doxorubicin (DXR), tautomycetin, and daptomycin biosynthesis in their natural host strains (Noh et al., 2010; Nah et al., 2012; Kim et al., 2014). However, WblA activates natamycin biosynthesis in *Streptomyces chattanoogensis* (Yu et al., 2014).

The third level is pathway-specific regulators (PSRs), which are taken as the master switches of antibiotic production. PSRs are situated in the BGCs of secondary metabolites and directly regulate the transcription of the biosynthetic genes. So they have been called the “cluster-situated regulators (CSRs).” SARP (*Streptomyces* antibiotic regulatory proteins), LAL (large ATP-binding regulators of the LuxR family) and PAS-LuxR family regulators usually belong to PSRs (Martin and Liras, 2012). The ActII-ORF4 and RedD of *S. coelicolor* and DnrI of *S. peucetius* are typical SARPs family activators (Song et al., 2011). The LAL family regulators, which comprise an N-terminal ATP/GTP-binding domain and a C-terminal LuxR family DNA-binding domain, usually activate the biosynthesis of antibiotic in *Streptomyces*. PimR, RapH, NysRI, AveR, and SlnR, which are typical LAL regulators, are located in type I polyketide BGCs in *Streptomyces* (Anton et al., 2004; Sekurova et al., 2004; Kuscer et al., 2007; Guo et al., 2010; Zhu et al., 2017). The whole process of avermectin biosynthesis is controlled by direct interaction between AveR and all promoters of *ave* cluster (Guo et al., 2010). The PAS-LuxR family regulator is characterized with an N-terminal PAS sensory domain and a C-terminal LuxR type DNA binding domain. PAS domain probably function as responsor to light, redox potential, oxygen, overall energy level of a cell, and small ligands (Taylor and Zhulin, 1999). For the cytosol localization, proteins containing PAS domains can sense internal signals and other environmental factors which cross the cell membrane. PimM, a PAS-LuxR family regulator, positively regulates pimaricin production in *Streptomyces natalensis* (Anton et al., 2007). The orthologs of PimM have been represented in most of the reported BGCs of antifungal polyketides like amphotericin (AmphRIV), candicidin (FscRI), nystatin (NysRIV), and filipin (PteF) gene clusters (Santos-Aberturas et al., 2011). PSRs also contain other family repressor or activator. The production of antibiotic can be positively or negatively regulated by TetR, MarR, LysR, and IclR family regulators (Molina-Henares et al., 2006; Martin and Liras, 2012; Cuthbertson and Nodwell, 2013; Zhang et al., 2015; Guo et al., 2018).

The fourth level is the feedback regulation which is brought by antibiotic and/or intermediates to coordinate antibiotic production and transport. Evidences has shown that antibiotic functions as signals to regulate the production of antibiotic besides as feedback substances for the enzymatic reactions. Antibiotic, as ligand for proper regulator, affects the final production in *Streptomyces*. The expression of antibiotic biosynthetic genes was modulated by the RedZ and undecylprodigiosin complex (Wang L. et al., 2009). The activity of AtrA, which regulates primary and secondary metabolism, is reduced by lidamycin of *Streptomyces globisporus* and actinorhodin (ACT) of *S. coelicolor* (Li et al., 2015). The biosynthesis of jadomycin is dynamically modulated by the interaction among jadomycin B, chloramphenicol, JadR1 and JadR2 in *Streptomyces venezuelae* (Wang L. et al., 2009; Xu D. et al., 2010). Daunorubicin (DNR) biosynthesis is regulated by three DNA binding regulatory proteins (DnrI, DnrN, and DnrO). The DNA binding activity of DnrO can be modulated by Rhodomycin D, a glycosylated precursor of DXR (Jiang and Hutchinson, 2006). Simocyclinone and its precursors inhibit the binding activity of SimReg1 to several promoter regions of simocyclinone biosynthesis genes and SimReg1 encoding gene (Horbal et al., 2012). As a GBL receptor-like protein, PapR5, which is the major regulator of pristinamycin biosynthesis, may sense pristinamycin or intermediate(s) of the pathway (Mast et al., 2015). SsaA can activate sansanmycin biosynthesis by binding to five different regions within the sansanmycin BGC. The sansanmycins A and H inhibit DNA-binding activity of SsaA in a concentration-dependent manner (Li et al., 2013). The rifamycin B, the end product of rifamycin biosynthesis, can relieve the repression of RifQ on the transcription of the rifamycin efflux pump (RifP) (Lei et al., 2018). Transporters may affect product maturation. Deletion of *nysG* and *nysH*, two ABC transporters encoding genes, resulted in ca. 35% reduction of nystatin production and accumulation of its deoxy precursor in *Streptomyces noursei*. NysGH complex is prone to export nystatin. Its activity would enhance the last biosynthetic step by relief of the feedback through final product removal (Sletta et al., 2005). ‘LanT,’ the dedicated ABC transporter for both class I and II lantibiotics, plays an important role in production of the final product (Gebhard, 2012).

Signals and regulators, dynamic fluctuating on the metabolic state of the cell and its environment, are extensively involved in regulation of secondary metabolism. The abundances of nitrogen, phosphate and carbon sources are nutrimental signal for the onset of secondary metabolism. It has been reported that GlnR and PhoR–PhoP was involved in the type of regulation depending on the carbon, nitrogen, and phosphate supply. For the similarity of binding sites, PhoP and GlnR showed competitive binding to target genes in some cases (Romero-Rodriguez et al., 2018). In *Saccharopolyspora erythraea*, the GlnR regulon is not only involved in nitrogen metabolism, but it also appears to control ABC-type transporters for uptake of carbon sources (Liao et al., 2015). In *S. avermitilis*, AveR positively regulates avermectin production and negatively affects oligomycin biosynthesis, respectively (Guo et al., 2010). AveI, an AtrA-like regulator, regulates production of avermectin, oligomycin, melanin, and morphological differentiation by directly regulating the transcription of *ave*, *olm*, *melC1C2*, *ssgRD*, *wblI* and genes in primary metabolism, including substrates transport, the metabolism of amino acids, lipids, and carbohydrates (Liu et al., 2019). Usually more than twenty secondary metabolic pathways exist in most of the *Streptomyces* (Nett et al., 2009). There is complex cross-talk regulation among different biosynthetic clusters and between the primary and secondary metabolism. With the deep understanding the regulatory mechanism of the antibiotic production, there is a consensus that the complicated and interweaved regulatory networks contribute to the dynamic antibiotic production in *Streptomyces*.

## Enhancement of Antibiotic Production in *Streptomyces* by Rewiring the Regulatory Networks

As mentioned above, the regulatory networks constitute major bottlenecks to over produce target antibiotics. The manipulation of regulatory genes can contribute to overcome these bottlenecks to turn on the expression of gene clusters for antibiotic production. Various strategies have been taken to manipulate regulatory genes to achieve the optimal antibiotic production in both the native and/or heterologous hosts.

### Enhancing Antibiotic Production by Overexpression of Positive Regulator Genes

The regulators also can be defined as positive and negative regulators according their effect on the antibiotic production. The positive regulators (activators) can promote the biosynthesis of antibiotics. But the negative ones (repressors) can repress the biosynthesis of antibiotics (Martin and Liras, 2010). Since the positive regulators activate the transcription of antibiotic BGCs, they can be manipulated to enhance the production of antibiotic in *Streptomyces*. The titer improvement can efficiently and simply be achieved by over-expression of genes encoding activators with proper promoters. As listed in Table 1, overexpression of genes encoding LAL family regulators, such as MilR, NemR, and AveR, has been used to increase production of milbemycin in *S. bingchenggensis* BC04, nemadectin in *S. cyaneogriseus* subsp. *non-cyanogenus* NMWT1 and avermectin in *S. avermitilis*, respectively (Guo et al., 2010; Zhang Y. et al., 2016; Li et al., 2019). Overproduction of nikkomycin has been achieved by engineering of the CSR activator gene *sanG* with different constitutive promoters (Liu et al., 2005). Overproduction of oxytetracycline (OTC) has been achieved by overexpression of the CSR activator gene *otcR* as tandem copies under the control of a constitutive SF14 promoter (Yin et al., 2015). Similar strategy has also been used to overproduce tacrolimus (FK506) in *Streptomyces tsukubaensis* NRRL18488 by overexpression of *bulZ* (Ma et al., 2018). Other examples include LysR family regulator for ascomycin production in *S. hygroscopicus* var. *ascomyceticus* (Song et al., 2017), PAS-LuxR family regulator for wuyiencin production in *Streptomyces wuyiensis* CK-15 (Liu et al., 2014), and Crp/Fnr family regulator for leinamycin production in *Streptomyces atroolivaceus* (Huang et al., 2016).

**TABLE 1:** Examples of antibiotic production enhancement in *Streptomyces* by regulatory gene manipulation.

**Strategy**	**Antibiotics**	**Strains**	**Regulators (family)**	**Yield**	**References**
Overexpression of positive genes	Milbemycin	*S. bingchenggensis*	MilR(LAL)	138%	Zhang Y. et al., 2016
	Nemadectin	*S. cyaneogriseus*	NemR(LAL)	179.9%	Li et al., 2019
	Avermectin	*S. avermitilis*	AveR(LAL)	164%	Guo et al., 2010
	Nikkomycin	*S. ansochromogenes*	SanG(SARP)	200%	Liu et al., 2005
	Oxytetracycline	*S. rimosus*	OtcR(SARP)	649%	Yin et al., 2015
	FK-506	*S. tsukubaensis*	BulZ (SARP)	∼330%	Ma et al., 2018
	Wuyiencin	*S. ahygroscopicus*	WysR(PAS-LuxR)	300%	Liu et al., 2014
	Leinamycin	*S. atroolivaceus*	LnmO(Crp/Fnr)	300%	Huang et al., 2016
	Pimaricin	*S. natalensis*	PimM(PAS-LuxR)	240%	Anton et al., 2007
	Pimaricin	*S. chattanoogensis*	ScnRII(PAS-LuxR)	400%	Du et al., 2009
	Milbemycin	*S. hygroscopicus*	MilR2 (TetR)	34.4%	Wei K. et al., 2018
	Avermectin	*S. avermitilis*	SAV4189 (MarR)	250%	Guo et al., 2018
	FK-506	*S. tsukubaensis*	FkbN (LAL)	176%	Zhang X. S. et al., 2016
	Daptomycin	*S. roseosporus*	DepR1(TetR)	141%	Yuan et al., 2016
	Daptomycin	*S. roseosporus*	DptR3(*MarR)*	131%	Zhang et al., 2015
Deletion of negative regulatory genes	Avermectin	*S. avermitilis*	SAV151(TetR)	200%	He et al., 2014
	Calcimycin	*S. chartreusis*	CalR3(TetR)	280%	Gou et al., 2017
	Pristinamycin	*S. pristinaespiralis*	PapR3(TetR)	240%	Meng et al., 2017
	Daptomycin	*S. roseosporus*	WblA	151%	Huang et al., 2017
	Pikromycin	*S. venezuelae*	WblA	350%	Woo et al., 2014
	Doxorubicin	*S. peucetius*	WblA	170%	Noh et al., 2010
	Platensimycin	*S. platensis*	PtmR1(GntR)	∼500%	Smanski et al., 2009
	Natamycin	*S. natalensis*	PhoRP(TCS)	180	Mendes et al., 2007
	Milbemycin	*S. bingchenggensis*	NsdA	150%	Wang X. J. et al., 2009
	Natamycin	*S*. *lydicus*	NsdA	190%	Wu et al., 2017
	Avermectin	*S. avermitilis*	AveI(AtrA)	1600%	Chen et al., 2008
	Nystatin A1	*S. ahygroscopicus*	TtmRIV(PAS-LuxR)	212%	Cui et al., 2015
	Rapamycin	*S. rapmycinicus*	RapS(TetR)	460%	Yoo et al., 2015
	Rapamycin	*S. rapmycinicus*	RapY(TetR)	370%	Yoo et al., 2015
Deletion of GBL receptors	Tylosin	*S. fradiae*	TylP	∼200%	Stratigopoulos et al., 2002
	Avermectin	*S. avermitilis*	AvaR1	∼300%	Wang et al., 2014
	Milbemycin	*S. bingchenggensis*	SbbR	125%	He et al., 2018
	Natamycin	*S. natalensis*	SngR	460%	Lee et al., 2005
	FK506	*S. tsukubaensis*	BulR1	27.8%	Salehi-Najafabadi et al., 2014
	Clavulanic acid	*S. clavuligerus*	Brp	300%	Santamarta et al., 2005
	Validamycin	*S. hygroscopicus*	ShbR1/R3	∼55%	Tan et al., 2015
Overexpression the feedback transporters	Avermectin	*S. avermitilis*	AvtAB	∼50%	Qiu et al., 2011
	Daunorubicin	*S. peucetius*	*DrrC*	510%	Malla et al., 2010
	Rifamycin	*A. mediterranei*	*RifQ*	200%	Lei et al., 2018
Ribosomal engineering	Avermectin	*S. avermitilis*	σ^hrdB^(A56 A393)	150%	Zhuo et al., 2010
	Actinorhodin	*S. lividans*	RpsL(K88E, L90K)	290%	Okamoto-Hosoya et al., 2003b
	Actinorhodin	*S. coelicolor*	K88E, the GI92	200%	Wang G. et al., 2009
	Salinomycin	*S. albus*	RpsL(K88R), RpoB	230%	Tamehiro et al., 2003
	A21978C	*S. roseosporus*	RpsL K43N	220%	Wang et al., 2012
	Chloramphenicol	S. coelicolor*	RpsL(K88E) RpoB(S433L)	∼1000%	Gomez-Escribano and Bibb, 2011

*Heterologous expression.

The expression level of positive regulator is not always correlated to the production of antibiotic. E.g., overexpression of *mil*R with a strong constitutive promoter led to decrease of milbemycin production in *S. bingchenggensis* (Zhang Y. et al., 2016). These showed that the threshold of the over-expressed regulator was key point to determine the production of antibiotic in *Streptomyces*.

### Enhancing Antibiotic Production by Removal of Repressor Genes

The TetR and LysR family regulators are widely distributed in the genome of *Streptomyces*. Most of TetR and LysR regulators function as repressors. More details of TetR family regulators have been reviewed elsewhere (Cuthbertson and Nodwell, 2013). Deletion of TetR family repressors has been used to increase avermectin production in *S. avermitilis* (He et al., 2014), calcimycin production in *Streptomyces chartreusis* NRRL 3882 (Gou et al., 2017), and pristinamycin production in *Streptomyces pristinaespiralis* (Meng et al., 2017).

WblA, a WhiB-like protein, also widely distributes among actinomycetes (Kim et al., 2014; Huang et al., 2017). WblA affects morphological development and antibiotic biosynthesis. It generally functions as a global repressor of antibiotic biosynthesis, such as DXR biosynthesis in *S. peucetius*, tautomycetin biosynthesis in *Streptomyces* sp. CK4412, and daptomycin biosynthesis in *Streptomyces roseosporus*. Deletion of *wblA* leads to overproduction of pikromycin in *S. venezuelae* (Woo et al., 2014), daptomycin in *S. roseosporus* (Huang et al., 2017). Other examples include deletion of genes encoding GntR family regulators for platensimycin and platencin overproduction in *Streptomyces platensis* (Smanski et al., 2009).

The *nsdA*, a gene negatively affecting *Streptomyces* differentiation, had been proved a pleiotropic negative regulatory gene in *S. coelicolor* (Li et al., 2006). It plays a negative role in sporulation, morphological differentiation and antibiotic synthesis. The overproduction of ACT, calcium-dependent antibiotic (CDA), and methylenomycin was detected in a *nsdA* mutant. The *nsdA* homologous genes have been found to conservatively distribute in *Streptomyces*. The milbemycin A4 and nanchangmycin production increased in *nsdA* mutant of *S. bingchengensis* by 1.5-fold and 9-fold, respectively (Wang X. J. et al., 2009). The natamycin production can be increased 1.9-fold by deletion of *nsdA* gene in *Streptomyces lydicus* A02 (Wu et al., 2017).

γ-Butyrolactone receptors, like the A-factor receptor ArpA, repress transcription of *adpA* to affect the production of streptomycin in *S. griseus*. Deletion of the GBL receptors probably can promote antibiotic production. The validamycin production of mutants with deletion of *arpA* homologs were increased by 26% (Δ*shbR1*) and 20% (Δ*shbR3*) in *S. hygroscopicus* 5008, respectively (Tan et al., 2015). Deletion of *avaR1*, the avenolide (a novel butenolide-type autoregulator) receptor encoding gene, increased production of avermectin B1a approximately 1.75 times compared with the parent strain in a high-producing *S. avermitilis* strain (Wang et al., 2014).

### Enhancing Antibiotic Production by Manipulation of Feedback and Transport

Genes encoding exporters, which are responsible for the secretion of antibiotic, often situate in their BGCs. Various BGC-linked transporters, belonging to ATP-binding cassette (ABC) superfamily and major facilitator superfamily (MFS) are responsible for secreting antibiotics. Pumping out of toxic end-products can achieve more durable and sustainable productivity.

It has been proved that the expression of BGCs was greatly affected by the secretion of end-products, even without toxicity. ActA (ActII-ORF2) and ActB (ActIIORF3), activate the transcription of BGCs in a feed-forward by transportation of the end-products (Tahlan et al., 2007; Xu et al., 2012). Only one fifth of ACT was produced by the *actAB* mutant. There are two waves for ACT production. The expression of key *act* genes is initially induced by an ACT biosynthetic intermediate. The ACT production is fully induced only when the inner ACT is pumped out.

Overexpression of AvtAB, an ABC transporter, enhance the Production of avermectin B1a with two-folds. But the production level of oligomycin A, another product from *S. avermitilis*, was found unaltered. The production promotion effects of *avtAB* could be specific to avermectin in *S. avermitilis* (Qiu et al., 2011). Co-overexpression of three OTC resistance genes, including *otrA* (encoding a ribosomal protection protein), *otrB* and *otrC* (encoding two efflux proteins), led to 179% increase of OTC production in *Streptomyces rimosus* M4018 (Yin et al., 2017).

The biosynthesis of BGCs for the actinobacterial ribosomally synthesized and posttranslationally modified peptides (RiPPs), like planosporicin and microbisporicin, is probably regulated in a feed-forward way.Their production and self-immunity is seemed to be modulated by the multiple ABC transporter genes in these BGCs (Foulston and Bibb, 2010; Sherwood et al., 2013). GouM, The MFS transporter, is responsible for the secretion of gougerotin outside of *Streptomyces graminearus* (Wei et al., 2014). The overexpression of BotT, a putative efflux pump encoded in the bottromycin BGC, increased bottromycin production about 20 times in a heterologous host (Huo et al., 2012).

Export of antibiotic is important for the producer to reduce the intracellular antibiotic concentration, which can relieve self-toxicity. In *Amycolatopsis mediterranei*, Δ*rifQ* mutant brought overexpression of RifP. The accelerated export of rifamycin may reduce the intracellular rifamycin concentration, relieve other possible feedback inhibition of rifamycin biosynthesis and finally lead to more than two-fold improvement of rifamycin B production (Lei et al., 2018). Overexpression of DrrC, which provide self-resistance to DNR and DXR, achieved 5.1-fold increase in DXR production in *S*. *peucetius* ATCC 27952 (Malla et al., 2010).

For there are many transporters help to transport end products or intermediates out of the cell, this can help to remove the feedback caused by the antibiotics or intermediates. Transporters enhance the efflux of the self-produced antibiotics, which can be an important strategy for self-protection from self-toxicity. Many antibiotic BGCs comprise functional exporter genes (Rees et al., 2009; Severi and Thomas, 2019). Also some exporters encoding genes for detoxification are outside of the BGCs. By systematical investigation, it has proved that more than 25 groups of genes contributed the efflux of molecules involved in natamycin biosynthesis (Shan et al., 2019).

### Enhancing Antibiotic Production by Ribosome Engineering

Classical strain improvement is usually achieved by multiple rounds of random mutagenesis and repetitively screening. Although the outcome is fruitful, the weakness of this strategy is time intensive and laborious. A ribosome engineering approach has been designed to obtain antibiotic overproducing strains by corelatively screening for mutants with proper drug resistant level, e.g., streptomycin resistance mutations (Hosoya et al., 1998). It has been proven to be an effective rational strategy for strain improvement. The percentage of overproducing mutants among drug resistance mutants can be up to 2–20% in a species dependent way, which is much higher than that of random mutagenesis. S12-mutated variants, which have mutations in the *rpsL* gene with resistance to streptomycin, stabilize the open A-site conformation and prevent streptomycin from binding (Holberger and Hayes, 2009). Screening of streptomycin resistant mutations has been demonstrated a useful tool for strain improvement in many *Streptomyces* strains (Ochi, 2007). The undecylprodigiosin production in *S. lividans* was activated in RpsL mutants with K88E, L90K, and R94G substitutions (Okamoto-Hosoya et al., 2003b). The industrial strain of *S. albus*, which had RpsL (K88R) and RpoB mutation, enhanced salinomycin production by 1.5-fold (Tamehiro et al., 2003). In *S. coelicolor*, over 10-fold ACT improved in RpsL (K88E and P91S) strains than the original strain (Okamoto-Hosoya et al., 2003a). Both the K88E mutation and an insertion mutation at GI92 led to substantially higher levels of ACT in *S. coelicolor* strains (Wang G. et al., 2009). Selection of mutations in the *rpsL* gene are widely used to improve the production in a variety of industrial antibiotic-producing strains (Tamehiro et al., 2003; Beltrametti et al., 2006).

Another method of “ribosome engineering” incorporates the inactivation of the *rsmG* gene, which encodes an *S*-adenosylmethionine (SAM)-dependent 16S rRNA methyltransferase. Recombinants with deletion of *rsmG* gene resulted in overproduction of ACT in *S. coelicolor* (Nishimura et al., 2007).

Previous reports showed that ribosome engineering was an effective strategy for yield improvement in *Streptomyces*. From the reference, ribosome engineering has been employed for yield improvement in more than fifty *Streptomyces* strains (Zhu et al., 2019). As a rational and cost-effective approach, ribosome engineering could be adapted to speed up strain development for those without clear genetic background. If compared with other direct genetic manipulation approach, the ribosome engineering still be with laborious screening to overcome the phenotype uncertainty of resistant mutants.

The above data show that manipulation of regulatory cascades can be an efficient way to improve the production of antibiotic. It is obvious that the balance and synergy between primary and secondary metabolism is very important for the overproduction of antibiotic. Rewiring regulatory network combined with metabolic engineering will be a more powerful way to enhance the production of antibiotic in *Streptomyces*.

## Discovering Novel Antibiotics Through Activating the Cryptic Gene Clusters by Unlocking Their Regulatory Network

The genome sequencing of streptomycetes demonstrated that each genome has the genetic capacity with 20–40 distinct gene clusters for secondary metabolites. They can produce far more compounds than reported previously (Nett et al., 2009). Since many of these gene clusters are expressed at low levels under laboratory conditions, they are called cryptic gene clusters. Several strategies have been designed to activate these clusters and discover novel molecules. The feasible approaches include uses of signals probes, ribosome engineering, regulatory unlocking and heterologous expression (Laureti et al., 2011; Ochi, 2017; Myronovskyi and Luzhetskyy, 2019). For this review is about the regulation in *Streptomyces*, the heterologous expression will not be discussed here.

### Activation of Cryptic Gene Clusters by Chemical or Physical Signals

Though *Streptomyces* has the potential to produce over 20 secondary metabolites, most BGCs are cryptic or silent. As we know from the regulatory cascades, there are many signaling factors affecting the expression of secondary metabolic gene, and many culture dependent methods have been developed (Yoon and Nodwell, 2014).

The one strain many compounds (OSMACs) approach has been previously applied to explore the secondary metabolic potential of different strains with altering a single parameter in the growth conditions or eliciting a stress response (Bode et al., 2002). Heat shock and ethanol shock are two widely applied measures. *S. venezuelae* produces negligible amounts of jadomycin when it is cultured at 27°C without heat/ethanol shock. The yield of jadomycin B reach 25 μg/ml after 12 h by shifting the temperature from 27 to 42°C. Similarly, cultures with 6% ethanol enhanced yields of jadomycin to as high as 30 μg/ml at 27°C (Doull et al., 1993, 1994). Another compound that is produced in response to the heat/ethanol shock is validamycin A (VAL-A) by *Streptomyces hygroscopicus*. VAL-A rapidly accumulated at relatively higher fermentation temperatures (37°C, 40°C, and 42°C). But the production was very low at lower temperatures (28°C, 30°C, 33°C, and 35°C) (Liao et al., 2009). Several other stress responses have been explored. New metabolites can be produced by *Streptomyces parvulus* upon increasing the hydrostatic pressure during fermentation (Bode et al., 2002). *S. coelicolor* can produce ectoine and 5-hydroxyectoine under high salt conditions and temperature stress (Bursy et al., 2008). Methylenomycin production can be activated in *S. coelicolor* by either alanine growth-rate-limiting conditions and/or acidic pH shock (Hayes et al., 1997).

As we know that the autoregulators can trigger the antibiotic production in *Streptomyces*. These classes of naturally produced chemical probes like GBLs can be used to elicit the expression of cryptic SBGs. Goadsporin, a compound isolated from *Streptomyces* sp. TP-A0584, was shown to stimulate the production of prodiginine antibiotic in *S. lividans*, and promote pigment production and morphogenesis on 36 streptomycetes (Onaka et al., 2001).

In *Streptomyces*, antibiotic production, which is usually coupled with the onset of development, is triggered by the interaction between DasR and the accumulation of GlcNAc after autolytic degradation of the vegetative mycelium. So GlcNAc can be used as a signal chemical to activate pathways for secondary metabolite biosynthesis. Several *Streptomyces* species were examined for their antibiotic production on MM plates (25 mM mannitol as the sole carbon source) with or without GlcNAc (50 mM). GlcNAc had a stimulating effect on antibiotic production on *S. clavuligerus*, *S. collinus*, *S. griseus*, *S. hygroscopicus*, and *S. venezuelae* (Rigali et al., 2008).

By investigating small molecules that perturb secondary metabolism, Justin group had screen out 19 compounds from 30,569 small molecules of the Canadian Compound Collection for their ability to alter the pigmentation of *S. coelicolor.* The ARC2 series (ARC2, ARC3, ARC4, and ARC5) were structurally related to triclosan, a synthetic antibiotic (Craney et al., 2012). Particularly, ARC2 altered the secondary metabolite output in all of the tested streptomycetes (Craney et al., 2012; Ahmed et al., 2013). These probes appear to have the potential to widely use as active elicitors for mining secondary metabolite.

Since *Streptomyces* is the soil dwelling microbe, it was reported that antibiotic overproduction could be promoted by 2- to 25-fold when the rare earth, scandium (Sc), added at a low concentration (10–100 mM) to cultures of *S. coelicolor* A3(2) (ACT producer), *Streptomyces antibioticus* (actinomycin producer), and *S. griseus* (streptomycin producer). Scandium was also effective in activating the dormant ability to produce ACT in *S. lividans* (Kawai et al., 2007). The rare earth elements, scandium and/or lanthanum, can activate the expression of nine genes belonging to nine secondary metabolite–BGCs by 2.5- to 12-fold in *S. coelicolor* A3(2). Several compounds can only be detected with HPLC in the rare earth-treated cultures (Tanaka et al., 2010).

Combined-culture is a co-culture method to activate secondary metabolism in *Streptomyces* (Onaka, 2017). The biosynthesis of red pigment by *S. lividans* TK23 can be influenced by co-culture with *Tsukamurella pulmonis* TP-B0596. It was proved that the biosynthesis of cryptic natural products in *Streptomyces* species can only be induced by living mycolic acid-containing bacteria (MACB). ∼90% of *Streptomyces* species, which isolated from soil samples collected in the Hokuriku district of Japan, show changes in secondary metabolism in combined-culture with *T. pulmonis* (Onaka et al., 2011).

Binding to a specific receptor, like the A factor, is proposed as possible mechanisms of activation of secondary metabolite biosynthesis by some chemical probes. The perturbation of host metabolism can be another reason to activate cryptic clusters. The ARC2 series, similar to triclosan, repress fatty acid biosynthesis by inhibiting the enoyl reductase FabI and change the flux of precursor molecules to antibiotic biosynthesis. Quorum sensing molecules could also important factors to induce the secondary metabolite production in actinomycetes. The results from co-cultivation probably due to physical cell to cell interactions, small molecule-mediated communication or metabolite precursor supply (Abdelmohsen et al., 2015; Jones et al., 2017). Though a great many of products are discovered through activation of cryptic gene clusters by chemical or physical signals, it still lacks clearly explanation of the molecular mechanism triggering the expression of the secondary metabolic genes. But for this approach is non-genetic dependent, it can be developed to high-throughput screening model to investigate more strains easily.

### Activation of the Cryptic Gene Clusters by Manipulation of the Regulatory Genes

The transcription processes of BGCs are usually modulated by specific regulatory gene clusters, including activators and repressors, which activate or repress biosynthesis, respectively. The identification of PSRs genes offers possibility to activate the desired BGCs either by inactivation of the repressor or over-expression of the activator genes (Baral et al., 2018).

TetR family of transcription regulators, which participate in the regulatory pathways associated with the efflux of antibiotic, cell–cell signaling, antibiotic biosynthesis, biofilm formation, etc., is one of the most predominant families of transcription factors in the prokaryotic system (Bhukya and Anand, 2017). The TetR family members usually serve as repressors which possess a N-terminal DNA-binding domain and a C-terminal signal reception domain (Cuthbertson and Nodwell, 2013). The inactivation of a TetR transcriptional repressor gene *arpRII* in *Streptomyces argillaceus* promoted the activation of the cryptic gene cluster *arp*, and discovery of argimycins P, a natural product in polyketide alkaloid family (Ye et al., 2018). ScbR, another TetR-like transcriptional repressor, has been confirmed to directly control the expression of the cryptic type I polyketide BGC by binding at two different positions of *kasO* promoter in *S. coelicolor* (Takano et al., 2005; Xu D. et al., 2010; Bhukya and Anand, 2017). GdmRIII, a TetR family transcriptional regulator, was connected with the biosynthetic pathways of geldanamycin and elaiophylin in *Streptomyces autolyticus* CGMCC0516 (Jiang et al., 2017). Moreover, it has been proved that the biosynthetic pathways including jadomycin, kinamycin, and auricin, were activated after removal of TetR family repressors, JadR/JadR2 (Zhang et al., 2013), AlpW (Bunet et al., 2011), and SCO1712 (Lee et al., 2010), respectively.

LuxR superfamily is another predominant class of regulators associated with quorum sensing (Baral et al., 2018). Contrary to the TetR family, LuxR-like proteins usually act as activators during secondary metabolic regulation. LuxR proteins consist of an N-terminal DNA binding domain and a C-terminal ligand-binding domain, where ligand binding induces homo-dimerization and subsequent binding of protein to DNA to initiate transcription (Maddocks and Oyston, 2008). LuxR proteins, which are abundant among Actinobacteria, Proteobacteria, and Firmicutes, play significant roles in improvement of antibiotic production and activation of cryptic gene clusters after overexpression. The LuxR family members, GdmRI and GdmRII, are positive regulators required for geldanamycin biosynthesis in *S. hygroscopicus* 17997, and inactivation of them resulted in blocked production of geldanamycin (He et al., 2008). The synthesis of β-lactam antibiotic thienamycin was first observed when ThnI protein of LuxR family was induced in *Streptomyces cattleya* (Rodriguez et al., 2008). Furthermore, the LAL–proteins are large ATP-binding regulators constituting a noteworthy branch of LuxR family (De Schrijver and De Mot, 1999). The LAL–subfamily members usually contain an additional ATP binding domain at the N-terminus, which is responsible for the interaction with the inducers, maltotriose and ATP (Danot, 2001; Baral et al., 2018). Series of studies have confirmed that LAL regulators play important roles in activating the silent gene clusters. Overexpression of LAL-type regulatory factor *samR* in *Streptomyces ambofaciens* activated a 150 kb cryptic gene cluster, and promoted the discovery of a glycosylated macrolide product with anti-cancer activity (Laureti et al., 2011). Similarly, LAL-type transcriptional regulators of GdmRI and GdmRII in *S. hygroscopicus* are positive to geldanamycin biosynthesis (Martin et al., 2019). Furthermore, the LAL-like 63-amino acid protein AfsS and its homologs have been shown to relate closely with the induction of avermectin in *S. avermitilis* (Lee et al., 2000), pikromycin in *S. venezuelae* (Maharjan et al., 2009), and DXR in *S. peucetius* (Parajuli et al., 2005).

Manipulation of the regulators in cryptic gene clusters probably the most convenient way to activate the pathway. But for the uncertainty of regulatory network, the success of activation is case by case.

### Activation the Cryptic Gene Clusters by Ribosome Engineering

Ribosome engineering, first proposed by Kozo Ochi, focuses on modifying ribosomal or RNA polymerases whose structural and functional changes related to the synthesis of secondary metabolites (Ochi et al., 2004). The introduction of specific mutations into the ribosome elements triggers structural changes to ribosome with affected protein synthesis, which ultimately influences secondary metabolism of the microorganism. Targeted strains are screened by mutations in antibiotic resistance acting on ribosome without special requirement of equipment or genetic background of the strains. Isoindolinomycin (Idm), an unprecedented bioactive polyketide with a novel isoindolinone-containing tetracyclic skeleton, was discovered by activation of cryptic gene through screening rifampicin-resistant (*rif*) mutants from actinobacteria strains (Thong et al., 2018). Piperidamycins production was induced in a gentamicin-resistant mutant of *Streptomyces mauvecolor*, whereas the wild strain does not produce these metabolites at any detectable level on various media (Hosaka et al., 2009; Baral et al., 2018). Ribosome engineering now is developed into an important technology for improving secondary metabolites with commercial value and stimulating new natural product production, and also contributes to increase in structural diversity of bioactive compounds. Mutations in these ribosomal genes have also been shown to activate the expression of silent or poorly expressed BGCs in *S. griseus* (Tanaka et al., 2009).

Most above strategies to activate cryptic gene clusters are by manipulating the original host. With developing of large DNA cloning, heterologous expression is extensively used to overexpress target clusters in super host strains (Yamanaka et al., 2014; Greunke et al., 2018; Choi et al., 2019). There are series of reviews on this aspect. Also some heterologous expression was solved with overexpression of regulatory genes by proper promoters in the target clusters (Fazal et al., 2019; Myronovskyi and Luzhetskyy, 2019; Nepal and Wang, 2019; Xu and Wright, 2019).

## Conclusion and Perspectives

**FIGURE 1:**
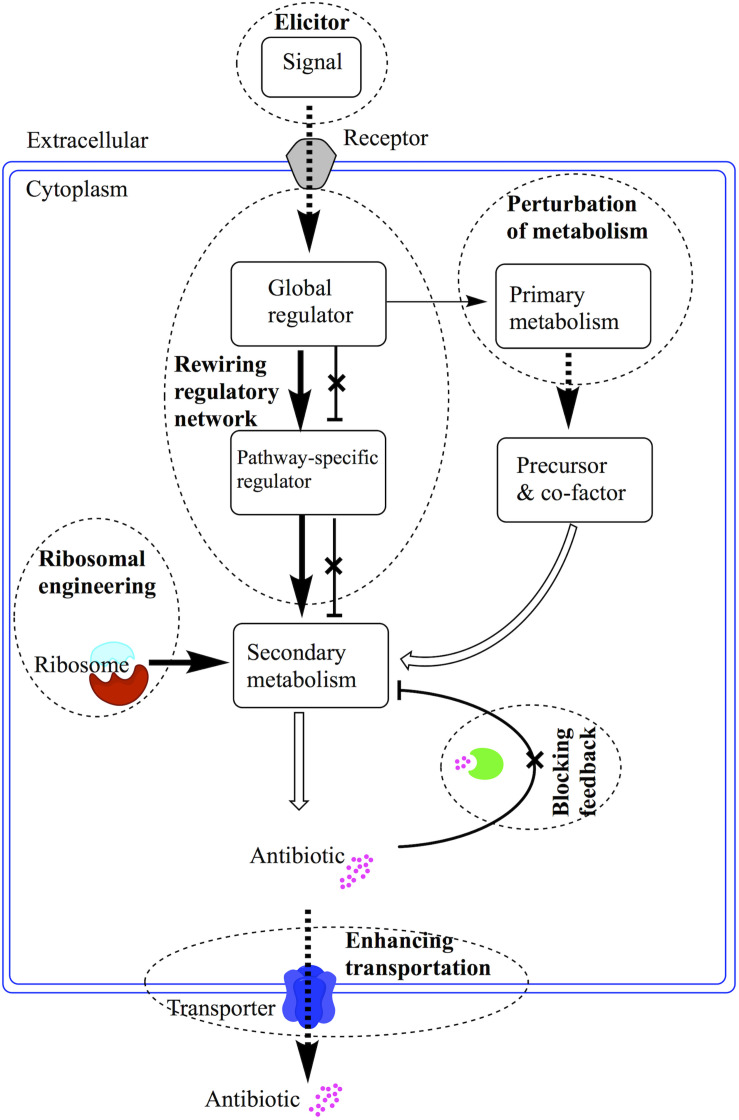
Schematic diagram of application strategies for regulatory cascades in *Streptomyces*. The regulatory cascades are illustrated with arrow linked rectangles. The dash circles are annotated with bold text to describe the strategies employed to enhance antibiotic production or discovery of novel antibiotics. The bold arrows mean overexpression of positive regulators. The crossed perpendicular lines mean the deletion of repressors or inhibition of feedback reactions. The dash bold arrows mean enhanced supply of precursor or efflux of antibiotics.

*Streptomyces*, which has complex morphological development and cell differentiation, can adapt to diverse environments by ingenious regulation and produce variety of secondary metabolites. The regulation of antibiotic production has been extensively investigated in the past years. For the complicated interactions among primary and secondary metabolic pathways, elucidation of the regulatory network in antibiotic production still was a tough task for scientist. With further recognizing the secondary metabolism regulatory cascades, novel proposed strategies can greatly promote the production enhancement and drug discovery in *Streptomyces*. For there are several reviews on the regulation of antibiotic biosynthesis, strategy to enhance production of antibiotic in *Streptomyces* and new compounds discovery in actinobacteria by Nodwell group and van Wezel group (van Wezel et al., 2009; Yoon and Nodwell, 2014; Rigali et al., 2018; van der Heul et al., 2018). Here we drafted a review on the cascades of regulation and its application in production enhancement and compound discovery (Figure 1). We proposed to give an extensive review on the regulation of antibiotic production in *Streptomyces*.

The complex hierarchical regulation constitutes a major bottleneck to over produce target antibiotic. On the basis of understanding the regulation of antibiotic biosynthesis, rewiring the regulatory network is much more efficient to optimize antibiotic producers than the classical random mutagenesis methods. It has mentioned above that the enhancement of antibiotic titers can be achieved by overexpression of positive regulator, inactivation of negative regulator, tuning feedback and ribosomal engineering. Combination of different strategies to manipulate regulatory genes can achieve higher antibiotic production in both the native and/or heterologous host. It is necessary to achieve maximal level of antibiotic production by systematically rewiring the regulatory network.

With the accumulation of explosive genome sequencing data, it has been estimated that *Streptomyces* species harbor a huge unexploited potential to produce novel natural products. A new discovery approach-microbial genome mining has been well developed in the past years (Baltz, 2019). How to efficiently activate cryptic gene clusters is still challenging to scientist. Because the interweaved regulatory networks in most cases are not well studied or understood. Elucidation of the regulatory network is crucial to discover new antibiotics.

For efficient manipulation of the regulatory genes, multi-*loci* simultaneous editing technology is necessary to be invented. The recent application of CRISPR-Cas9 dependent serials genome editing system provides a new opportunity for rapid rewiring regulatory network in *Streptomyces* (Tong et al., 2019). For precisely tuning the transcription level of regulators, a panel of quantitative promoters should be defined in *Streptomyces*. This would be practical for tuning the expression of secondary metabolic pathway. Though there are many publications on promoter engineering in *Streptomyces*. Still serial quantitative promoters are necessary for predictable engineering with synthetic biology strategy in future.

## Author Contributions

HX, XL, ZL, and XZ prepared material for the manuscript. HX, XM, and YL wrote the manuscript. All authors read and approved the final manuscript.

## Conflict of Interest

The authors declare that the research was conducted in the absence of any commercial or financial relationships that could be construed as a potential conflict of interest.
