# Corrigendum: The Application of Regulatory Cascades in *Streptomyces*: Yield Enhancement and Metabolite Mining

**DOI:** 10.3389/fmicb.2020.614274

**Published:** 2021-02-03

**Authors:** Haiyang Xia, Xiaofang Li, Zhangqun Li, Xinqiao Zhan, Xuming Mao, Yongquan Li

**Affiliations:** ^1^Institute of Biopharmaceuticals, Taizhou University, Taizhou, China; ^2^Institute of Pharmaceutical Biotechnology, School of Medicine, Zhejiang University, Hangzhou, China

**Keywords:** antibiotic production, regulatory cascades, rewiring regulatory network, unlocking cryptic metabolites, *Streptomyces*

In the original article, there was some errors. A portion of this text was reproduced from another article, this has now been rephrased and appropriately attributed.

A correction has been made to **The Regulatory Cascades of Antibiotic Production in *Streptomyces***, **paragraph 5**:

The fourth level is the feedback regulation which is brought by antibiotic and/or intermediates to coordinate antibiotic production and transport. Evidence has shown that antibiotic functions as signals to regulate the production of antibiotic besides as feedback substances for the enzymatic reactions. Antibiotic, as ligand for proper regulator, affects the final production in Streptomyces. The expression of antibiotic biosynthetic genes was modulated by the RedZ and undecylprodigiosin complex (Wang et al., 2009). The activity of AtrA, which regulates primary and secondary metabolism, is reduced by lidamycin of *Streptomyces globisporus* and actinorhodin (ACT) of *S. coelicolor* (Li et al., 2015). The biosynthesis of jadomycin is dynamically modulated by the interaction among jadomycin B, chloramphenicol, JadR1 and JadR2 in *Streptomyces venezuelae* (Wang et al., 2009; Xu et al., 2010). Daunorubicin (DNR) biosynthesis is regulated by three DNA binding regulatory proteins (DnrI, DnrN, and DnrO). The DNA binding activity of DnrO can be modulated by Rhodomycin D, a glycosylated precursor of DXR (Jiang and Hutchinson, 2006). Simocyclinone and its precursors inhibit the binding activity of SimReg1 to several promoter regions of simocyclinone biosynthesis genes and SimReg1 encoding gene (Horbal et al., 2012). As a GBL receptor-like protein, PapR5, which is the major regulator of pristinamycin biosynthesis, may sense pristinamycin or intermediate(s) of the pathway (Mast et al., 2015). SsaA can activate sansanmycin biosynthesis by binding to five different regions within the sansanmycin BGC. The sansanmycins A and H inhibit DNA-binding activity of SsaA in a concentration-dependent manner (Li et al., 2013). The rifamycin B, the end product of rifamycin biosynthesis, can relieve the repression of RifQ on the transcription of the rifamycin efflux pump (RifP) (Lei et al., 2018). Transporters may affect product maturation. Deletion of *nysG* and *nysH*, two ABC transporters encoding genes, resulted in ca. 35% reduction of nystatin production and accumulation of its deoxy precursor in *Streptomyces noursei*. NysGH complex is prone to export nystatin. Its activity would enhance the last biosynthetic step by relief of the feedback through final product removal (Sletta et al., 2005). ‘LanT,’ the dedicated ABC transporter for both class I and II lantibiotics, plays an important role in production of the final product (Gebhard, 2012).

A correction has been made to **Enhancing Antibiotic Production by Overexpression of Positive Regulator Genes**, **paragraph 1**:

The regulators also can be defined as positive and negative regulators according their effect on the antibiotic production. The positive regulators (activators) can promote the biosynthesis of antibiotics. But the negative ones (repressors) can repress the biosynthesis of antibiotics (Martin and Liras, 2010). Since the positive regulators activate the transcription of antibiotic BGCs, they can be manipulated to enhance the production of antibiotic in *Streptomyces*. The titer improvement can efficiently and simply be achieved by over-expression of genes encoding activators with proper promoters. As listed in **Table 1**, overexpression of genes encoding LAL family regulators, such as MilR, NemR, and AveR, has been used to increase production of milbemycin in *S. bingchenggensis* BC04, nemadectin in *S. cyaneogriseus* subsp. *non-cyanogenus* NMWT1 and avermectin in *S*. *avermitilis*, respectively (Guo et al., 2010; Zhang et al., 2016; Li et al., 2019). Overexpression of *sanG*, a CSR activator encoding gene, led to improvement of nikkomycin production (Liu et al., 2005). Tandem copies of *otcR* (a CSR activator gene), whose expression is driven by the SF14 promoter, can greatly enhance the production of oxytetracycline (OTC) (Yin et al., 2015). There are many examples in similar strategies to improve antibiotic production in *Streptomyces*. Overexpression of *bulZ, fkbR1, wysR*, and *lnmO* led to overproduction of tacrolimus (FK506), ascomycin, wuyiencin, and leinamycin, respectively (Liu et al., 2014; Huang et al., 2016; Song et al., 2017; Ma et al., 2018).

A correction has been made to **Enhancing Antibiotic Production by Manipulation of Feedback and Transport**, **Paragraph 1**:

Genes encoding exporters, which are responsible for the secretion of antibiotic, often situate in their BGCs. Various BGC-linked transporters, belonging to ATP-binding cassette (ABC) superfamily and major facilitator superfamily (MFS) are responsible for secreting antibiotics. Pumping out of toxic end-products can achieve more durable and sustainable productivity.

In the section **Enhancing Antibiotic Production by Manipulation of Feedback and Transport**, **Paragraphs 2, 3, and 4** should be replaced with the following text;

It has been proved that the expression of BGCs was greatly affected by the secretion of end-products, even without toxicity. ActA (ActII-ORF2) and ActB (ActIIORF3), activate the transcription of BGCs in a feed-forward by transportation of the end-products (Tahlan et al., 2007; Xu et al., 2012). Only one fifth of ACT was produced by the *actAB* mutant. There are two waves for ACT production. The expression of key *act* genes is initially induced by an ACT biosynthetic intermediate. The ACT production is fully induced only when the inner ACT is pumped out.

Overexpression of AvtAB, an ABC transporter, enhance the production of avermectin B1a with two-folds. But the production level of oligomycin A, another product from *S. avermitilis*, was found unaltered. The production promotion effects of *avtAB* could be specific to avermectin in *S. avermitilis* (Qiu et al., 2011). Co-overexpression of three OTC resistance genes, including *otrA* (encoding a ribosomal protection protein), *otrB* and *otrC* (encoding two efflux proteins), led to 179% increase of OTC production in *Streptomyces rimosus* M4018 (Yin et al., 2017).

The biosynthesis of BGCs for the actinobacterial ribosomally synthesized and posttranslationally modified peptides (RiPPs), like planosporicin and microbisporicin, is probably regulated in a feed-forward way. Their production and self-immunity is seemed to be modulated by the multiple ABC transporter genes in these BGCs (Foulston and Bibb, 2010; Sherwood et al., 2013). GouM, the MFS transporter, is responsible for the secretion of gougerotin outside of *Streptomyces graminearus* (Wei et al., 2014). The overexpression of BotT, a putative efflux pump encoded in the bottromycin BGC, increased bottromycin production about 20 times in a heterologous host (Huo et al., 2012).

The authors apologize for these errors and state that this does not change the scientific conclusions of the article in any way. The original article has been updated.
